# Does preoperative gabapentin or intraoperative ketorolac influence postoperative pain following hip arthroscopy?

**DOI:** 10.1093/jhps/hnad031

**Published:** 2023-10-31

**Authors:** Alex M Meyer, Krit Petrachaianan, Natalie A Glass, Robert W Westermann

**Affiliations:** Department of Orthopedics & Rehabilitation, University of Iowa, 200 Hawkins Drive, Iowa City, IA 52242, USA; Department of Orthopedics & Rehabilitation, University of Iowa, 200 Hawkins Drive, Iowa City, IA 52242, USA; Department of Orthopedics & Rehabilitation, University of Iowa, 200 Hawkins Drive, Iowa City, IA 52242, USA; Department of Orthopedics & Rehabilitation, University of Iowa, 200 Hawkins Drive, Iowa City, IA 52242, USA

## Abstract

Optimization of perioperative analgesia has important implications for patient satisfaction and short-term outcomes. This study’s purpose is to assess if preoperative gabapentin or intraoperative ketorolac influences postoperative pain or time to discharge following hip arthroscopy. In total, 409 patients who underwent hip arthroscopic femoroplasty and/or acetabuloplasty with a single surgeon for femoroacetabular impingement were retrospectively reviewed (September 2017 to February 2021). The effect of preoperative gabapentin or intraoperative ketorolac on postoperative visual analog scale (VAS) pain scores, perioperative opioids in morphine milligram equivalents (MMEs), time in post-anesthesia care unit (PACU), second-stage recovery and time to discharge was assessed using unadjusted and adjusted *t*-tests, and generalized linear models controlling for operative time, traction time, preoperative MME, intraoperative MME and postoperative MME were compared between the groups of gabapentin to no gabapentin and ketorolac to no ketorolac. There was no difference in first PACU VAS pain score, final PACU VAS score, VAS pain score prior to discharge, average VAS pain score or pain level on follow-up call in the unadjusted or adjusted analysis for the preoperative gabapentin or intraoperative ketorolac groups. Females had higher first PACU VAS pain score (6.05 versus 5.15 *P* = 0.0026), final PACU VAS pain score (4.43 versus 3.90, *P* = 0.0045), final VAS pain score prior to discharge (3.87 versus 3.03, *P* < 0.001) and average postoperative pain score (4.60 versus 4.03, *P* < 0.001), but no difference in VAS pain score on follow-up call following surgery. Gabapentin or ketorolac was not associated with decreased VAS pain scores or time to discharge after hip arthroscopy.

## INTRODUCTION

Hip arthroscopy is becoming a much more common surgery with an increase of 727% in the United Kingdom over the past decade [1]. Hip arthroscopy can be a surgical treatment to many conditions including femoroacetabular impingement (FAI), labral tears, loose bodies and snapping iliopsoas tendon. With the increase in the number of hip scopes being performed, there have been many attempts to determine optimal perioperative pain management including peripheral nerve blocks, local anesthetic injection and celecoxib [1]. Other studies have found that femoral nerve blocks or lumbar plexus blocks provided adequate analgesia but had an increase in fall rates following surgery [2]. Newer techniques such as pericapsular nerve group blocks [3], quadratus lumborum blocks [4] and fascia iliaca blocks [4] are becoming more widespread; however, they can be technically challenging and carry inherent risk [4]. The risks of peripheral nerve blocks include vascular injury, bleeding, nerve damage, local anesthetic systemic toxicity and quadriceps weakness [5]. Nerve blocks are also challenging due to the fact that the hip joint has multiple sensory innervations including the femoral and obturator nerves to the anterior hip capsule, while the posterior capsule is supplied by branches of sciatic nerve, nerve to quadratus femoral muscle and superior gluteal nerve. Additionally, the portal sites are in the lateral femoral cutaneous nerve distribution [6]. Therefore, we are investigating other less invasive methods of providing adequate analgesia in an effort to make the surgical experience more comfortable for patients to minimize opioid use and time spent in the hospital in order to facilitate greater patient safety and faster discharges.

Inadequately controlled pain has undesirable physiological and psychological consequences such as postoperative morbidity, delayed recovery, a delayed return to normal daily living and reduced patient satisfaction [7]. Other options include local anesthetic injected at the conclusion of the case, which has been shown to be just as effective as fascia iliaca blocks [8], or cold therapy, which was shown to have no difference compared to a regional block [9].

Opioids are commonly part of a multimodal analgesic plan. However, opioids also have risks such as nausea, respiratory depression and risk of physical dependence. Kessler *et al.* found that 98.6% of surgical patients receive opioids for postoperative pain and 13.6% of them experienced nausea, vomiting, allergic reactions, constipation, urinary retention, postoperative ileus, pruritis, sedation, dizziness, decreased coordination or decreased cognitive function [10].

Gabapentin has been used for perioperative analgesia for other procedures with reliable results, but it has not been shown to be efficacious for orthopedic procedures. For example, Mahoori *et al.* demonstrated that preoperative gabapentin was shown to decrease visual analog scale (VAS) pain scores and amount of opioid use in the first 24 h following hernia repair [11]. While Ul Huda *et al.* showed in a systematic review of premedication with gabapentin prior to shoulder arthroscopy a decrease in postoperative nausea and vomiting but no decrease in postoperative pain [12]. For hip procedures, two studies found that it did not improve postoperative pain after hip arthroplasty [13, 14], but there are currently no studies evaluating gabapentin for hip arthroscopy.

Nonsteroidal anti-inflammatory drugs (NSAIDs) are another potential medication to aid in analgesia following hip arthroscopy. NSAIDs reduce the amount of prostaglandins by inhibiting cyclooxygenase enzymes. Zhang *et al.* demonstrated less opioid use in the immediate 24-h postoperative period and lower pain scores after celecoxib administration when compared to placebo following hip arthroscopy for FAI [15]. While Kahlenberg *et al.* reported a statistically but not clinically significant decrease in time to discharge and decreased reported pain scores after administration of celecoxib with no change in opioid use following hip arthroscopy [16]. Cunningham *et al.* showed that ketorolac slightly decreases the amount of oral morphine milliequivalents but was not found to decrease the time in post-anesthesia care unit (PACU) or pain reported following hip arthroscopy for FAI [17]. If a benefit can be found through this study, gabapentin and/or ketorolac could be more widely used in an effort to decrease opioid use following surgery and decrease recovery times in postoperative areas.

Finally, postoperative pain is a complex issue, and as Munsch *et al.* described, the amount of pain a patient experiences and the need for opioid medication may depend largely on patient factors. They found that prior opioid use, age, mental health conditions and worker’s compensation cases had a large impact on postoperative opioid use [9]. Currently, at our institution, analgesic medications are determined by anesthesia staff depending on their personal preferences. With the increase in hip arthroscopy volume, we are looking for more definitive research on how to best treat pain following hip arthroscopy. The aims of this study include (i) identifying the effects that preoperative gabapentin administered in the preoperative area has on postoperative pain following hip surgery, (ii) identifying the effects of intraoperative intravenous ketorolac on postoperative pain and (iii) determining if these medications influence time in postoperative recovery areas. Our hypothesis was that preoperative gabapentin or intraoperative ketorolac will decrease postoperative VAS pain scores throughout the patient’s stay in the PACU, in second-stage recovery area and on follow-up call. We also hypothesized that preoperative gabapentin or intraoperative ketorolac will decrease the amount of opioid medications used in the PACU and second-stage recovery areas. Finally, we hypothesize that preoperative gabapentin or intraoperative ketorolac will decrease time in PACU and second-stage recovery areas, thus decreasing the time to discharge.

## METHODS

### Patients

**Fig. 1.**
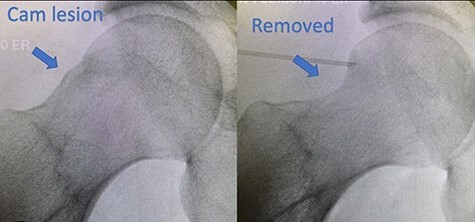
Left image: FAI with large cam; right image: status post-arthroscopic osteochondroplasty.

Approval for this study was granted by the Institutional Review Board. The data underlying this article will be shared on reasonable request to the corresponding author. This study was a retrospective review of our institution’s prospectively collected hip preservation registry. All patients who underwent hip arthroscopy from September 2017 to February 2021 were enrolled in the hip preservation registry. In the selected time frame, there were 490 patients who underwent hip arthroscopy at a single institution with a single fellowship trained orthopedic surgeon. Inclusion criteria included primary arthroscopic hip femoroplasty and/or acetabuloplasty for FAI with or without labral repair (Fig. 1). All other procedures such as psoas tendon release, loose body removal, isolated labral repair, Iliotibial (IT) band lengthening, Greater trochanter (GT) bursectomy gluteus medius repair, Anterior inferior illiac spine (AIIS) decompression through osteoplasty and septic hip irrigation and debridement were excluded. These criteria left 409 patients in the study population. All hip arthroscopy procedures were performed in the supine post-less position under general anesthesia. The central compartment was accessed first, and an inter-portal capsulotomy was used in all cases. At the conclusion of each procedure, an intra-articular and periportal injection of 20 mL of 0.75% ropivacaine was administered as 15 mL intra-articular and 5 mL in the portal sites.

### Data sources

Data including surgical procedure, operative time and amount, if any, of gabapentin administered preoperatively, amount, if any, of ketorolac that was given intraoperatively, duration of surgery, time in Stage 1 of PACU recovery, time in Stage 2 of recovery, total time to discharge, first VAS pain scale in PACU, last VAS pain scale, average VAS during PACU, pain during first follow-up phone call, pain at first postoperative clinic follow-up and amount of opioid medications administered following surgery were recorded. Nursing staff recorded the first VAS pain scale as soon as the patient was transferred from the operating room to the PACU area. Nursing staff recorded the last VAS pain scale just prior to transfer from PACU to second-stage recovery. The follow-up phone call was performed by clinic staff on the first business day following the procedure. Finally, the first postoperative clinic follow-up was typically 3 weeks after surgery.

### Treatment protocol

If the patient received gabapentin, it was administered by mouth in capsule form by anesthesia providers in the preoperative area before going to the operating room. Of the 372 patients who received gabapentin, a majority (366) received either 300 or 600 mg of gabapentin. Three participants under the age of 18 years received 100 or 150 mg, and three participants received 900 or 1000 mg of gabapentin. If a patient received ketorolac, it was administered by anesthesia providers through the intravenous route just prior to emergence from anesthesia immediately following the completion of the procedure. A majority of patients (166) who received ketorolac received 30 mg of ketorolac. However, 13 patients received between 10 and 27 mg of ketorolac dependent on weight-based dosing. The medications that the patient received were at the discretion of anesthesia providers. Therefore, patients could receive gabapentin, ketorolac, neither or both. Depending on the medications received, they were included in the respective analysis. Preoperative opioids were administered in the preoperative holding area, intraoperative opioids were given intravenously in the operating room and postoperative opioids were given in the PACU or second-stage recovery areas. No patients received nerve blocks. The duration data were obtained from the operating room records. The VAS scores were reported by nursing or anesthesia and documented in the patient’s chart and reviewed by the research team.

### Statistical analysis

All 409 patients were grouped according to receiving (*n* = 372) or not receiving preoperative gabapentin (*n* = 37) (Table I). The same 409 patients were grouped according to receiving (*n* = 179) or not receiving intraoperative ketorolac (*n* = 230) (Table IV). Groups were evaluated for significant differences in preoperative morphine milligram equivalents (MMEs), intraoperative MME, postoperative MME and total MME between each group in unadjusted analyses using *t*-tests and analyses adjusted for operative time, traction time, preoperative MME, intraoperative MME and postoperative MME using generalized linear models.

**Table I.** Demographic data of the patients who received preoperative gabapentin compared to the group that did not receive preoperative gabapentin.

*Gabapentin*							
	*No preoperative gabapentin*			*Preoperative gabapentin*			
*Variable*	*n*	*Mean*	*Standard error*	*n*	*Mean*	*Standard error*	*P*
Age (years)	37	27.41	12.05	371	26.22	10.53	0.5192
Height (cm)	37	170.13	11.60	372	171.48	10.15	0.4459
Weight (kg)	37	78.73	25.50	372	78.04	19.30	0.8413
BMI (kg/m^2^)	37	26.89	7.05	372	26.27	5.25	0.5065
Gender (female)	25	60%		221	61%		
Preoperative alpha angle	30	66.30°	10.95	287	68.65°	10.35	0.2404
Preoperative lateral center edge angle	30	30.83°	6.58	287	30.68°	6.59	0.9078
Preoperative Tonnis angle	30	6.22°	3.912	287	6.40°	3.65	0.2665
Operative time (min)	37	93.19	30.48	372	93.52	23.38	0.9368
Traction time (min)	29	43.07	11.18	350	43.65	11.15	0.7882
Preoperative gabapentin (mg)	37	0.00	0.00	372	469.76	156.89	<0.001
Intraoperative ketorolac (mg)	37	7.91	13.22	372	13.28	14.73	0.0333

*P*-value <0.05 is in **Bold**.

### Power analysis

#### Gabapentin group

In order to achieve 80% power to reject the null hypothesis of equal means when the population mean difference is 1 point on the VAS pain scale with standard deviations of 1.74 for Group 1 and 6.87 for Group 2, and with a significance level (alpha) of 0.05 using a two-sided two-sample unequal variance *t*-test, group sample sizes would need to be 396 in each group. If there was an expected difference of 2 points on the VAS pain scale, the sample size needed is 101 per group.

#### Ketorolac group

In order to achieve 80% power to reject the null hypothesis of equal means when the population mean difference is 1 point on the VAS pain scale with standard deviations of 1.66 for Group 1 and 2.79 for Group 2, and with a significance level (alpha) of 0.05 using a two-sided two-sample unequal variance *t*-test, group sample sizes would need to be 84 in each experimental group. If there was an expected difference of 2 points on the VAS pain scale, the sample size needed is 22 per group.

## RESULTS

### Gabapentin

There were 37 participants who did not receive gabapentin and 372 who did receive preoperative gabapentin. The groups were similar in age, height, weight, body mass index (BMI), sex, radiographic measurements, operative time and traction time (all *P* > 0.05, Table I). The group that received gabapentin received an average dose of 469.67 mg. They also received on average a higher dose of ketorolac (13.28 versus 7.81 mg, *P* = 0.0333). In the unadjusted analysis, the participants who received gabapentin also received more preoperative MME (gabapentin 6.84, no gabapentin 3.14, *P* < 0.001) (Table II). In the analysis adjusted for operative time, traction time, preoperative MME, intraoperative MME and postoperative MME, participants who received preoperative gabapentin had no statistical between-group difference in time in PACU, time in second-stage recovery, time to discharge, first PACU VAS pain score, final PACU VAS pain score, final VAS pain score prior to discharge, average VAS pain score and pain level on follow-up call (all *P* > 0.05, Table III).

**Table II.** Unadjusted analysis for experimental groups receiving gabapentin compared to no gabapentin.

*Gabapentin*	*Unadjusted analyses*				
	*Gabapentin− (n = 37)*		*Gabapentin+ (n = 372)*		
*Response variable*	*Mean*	*Standard error*	*Mean*	*Standard error*	*P*
Time in PACU (min)	102.51	7.00	102.16	10.14	0.9621
Time in second-stage recovery (min)	87.83	6.51	81.31	9.44	0.3402
Time from PACU to discharge (min)	192.81	9.77	183.58	14.15	0.3684
Preoperative MMEs	3.14	0.68	6.84	0.98	0.0000
Intraoperative MMEs	30.03	2.02	27.92	2.93	0.3221
Postoperative MMEs	24.67	1.93	25.19	2.80	0.7960
Total MMEs	57.84	2.84	59.96	4.11	0.4757
First PACU VAS pain score	5.35	0.48	5.73	0.70	0.4493
Final PACU VAS pain score	4.20	0.30	4.24	0.44	0.8918
Final VAS pain score prior to discharge	3.72	0.29	3.55	0.42	0.5671
Average VAS pain score	4.45	0.27	4.51	0.40	0.8254
Pain level on follow-up call	2.79	0.34	2.60	0.49	0.5829

*P*-value <0.05 is in **Bold**.

**Table III.** Multivariate regression models for experimental variables after controlling for operative time, traction time, preoperative MME, intraoperative MME and postoperative MME for the gabapentin group compared to the non-gabapentin group.

*Gabapentin: adjusted for operative time, traction time, preoperative MME, intraoperative MME and postoperative MME*					
	*Gabapentin− (n = 37)*		*Gabapentin+ (n = 372)*		
*Response variable*	*Mean*	*Standard error*	*Mean*	*Standard error*	*P*
Time in PACU (min)	80.27	18.64	79.83	20.33	0.9567
Time in second-stage recovery (min)	74.66	19.55	68.33	21.30	0.4554
Time from PACU to discharge (min)	165.40	27.46	156.83	29.92	0.4715
First PACU VAS pain score	5.93	1.36	6.84	1.49	0.1260
Final PACU VAS pain score	4.00	0.88	4.24	0.96	0.5219
Final VAS pain score prior to discharge	4.24	0.85	3.87	0.93	0.3276
Average VAS pain score	4.64	0.78	4.90	0.85	0.4469
Pain level on follow-up call	2.26	1.02	2.30	1.11	0.9225

Means are predicted values evaluated with covariates at 0.

*P*-value <0.05 is in **Bold**.

### Ketorolac

There were 230 participants who did not receive intraoperative ketorolac and 179 who did receive intraoperative ketorolac. The groups were similar in age, height, weight, BMI, sex, radiographic measurements, operative time and traction time (all *P* > 0.05, Table IV). The group that received ketorolac on average received a dose of 29.24 mg. They also received on average a larger dose of gabapentin (455.59 versus 405.22, *P* = 0.0120). Unadjusted analysis showed that those who received ketorolac received more intraoperative MME (ketorolac 31.08, no ketorolac 25.80, *P* < 0.001) and total MME (ketorolac 63.10, no ketorolac 57.18, *P* = 0.0005) (Table V). After controlling for operative time, traction time, preoperative MME, intraoperative MME and postoperative MME, the ketorolac and no ketorolac groups did not differ in time in PACU *t*, time in second-stage recovery, time to discharge, first PACU VAS pain score, final PACU VAS pain score, final VAS pain score prior to discharge, average VAS pain score, pain level on follow-up call, preoperative MME, intraoperative MME and postoperative MME (all *P* > 0.05, Table VI).

**Table IV.** Demographic data of the patients who received intraoperative ketorolac compared to the patients who did not get intraoperative ketorolac.

*Ketorolac*							
	*No preoperative ketorolac*			*Preoperative ketorolac*			
*Variable*	*n*	*Mean*	*Standard error*	*n*	*Mean*	*Standard error*	*P*
Age (years)	229	26.66	10.85	179	25.89	10.45	0.4701
Height (cm)	230	170.73	10.15	179	172.17	10.43	0.1601
Weight (kg)	230	78.04	19.56	179	78.19	20.39	0.9394
BMI (kg/m^2^)	230	26.60	5.49	179	25.97	5.34	0.2483
Gender (female)	136	59%		115	64%		
Preoperative alpha angle	181	68.24°	11.06	136	68.68°	9.52	0.7109
Preoperative lateral center edge angle	181	30.47°	6.94	136	30.98°	6.10	0.5032
Preoperative Tonnis angle	181	5.61°	3.87	136	6.90°	3.31	0.3834
Operative time (min)	230	93.96	23.63	179	92.89	24.66	0.6566
Traction time (min)	210	43.20	10.70	169	44.10	11.68	0.4373
Preoperative gabapentin (mg)	230	405.22	208.49	179	455.59	188.87	0.0120
Intraoperative ketorolac (mg)	230	0.00	0.00	179	29.24	3.08	<0.001

*P*-value <0.05 is in **Bold**.

**Table V.** Unadjusted analysis for experimental groups receiving ketorolac compared to no ketorolac.

*Ketorolac*	*Unadjusted analyses*				
	*Ketorolac− (n = 230)*		*Ketorolac+ (n = 179)*		
*Response variable*	*Mean*	*Standard error*	*Mean*	*Standard error*	*P*
Time in PACU (min)	103.41	2.81	100.64	5.09	0.5131
Time in second-stage recovery (min)	84.29	2.60	78.79	4.72	0.1632
Time from PACU to discharge (min)	188.94	3.88	178.51	7.05	0.0773
Preoperative MMEs	6.25	0.28	6.84	0.51	0.1653
Intraoperative MMEs	25.80	0.79	31.08	1.44	<0.0001
Postoperative MMEs	25.12	0.78	25.18	1.41	0.9617
Total MMEs	57.18	1.12	63.10	2.03	0.0005
First PACU VAS pain score	5.81	0.19	5.55	0.35	0.3738
Final PACU VAS pain score	4.30	0.12	4.16	0.22	0.4723
Final VAS pain score prior to discharge	3.49	0.11	3.65	0.21	0.3560
Average VAS pain score	4.54	0.11	4.46	0.20	0.6344
Pain level on follow-up call	2.60	0.13	2.64	0.24	0.8280

*P*-value <0.05 is in **Bold**.

**Table VI.** Multivariate regression models for experimental variable after controlling for operative time, traction time, preoperative MME, intraoperative MME and postoperative MME for the ketorolac group compared to the non-ketorolac group.

*Ketorolac: adjusted for operative time, traction time, preoperative MME, intraoperative MME and postoperative MME*					
	*Ketorolac− (n = 230)*		*Ketorolac+ (n = 179)*		
*Response variable*	*Mean*	*Standard error*	*Mean*	*Standard error*	*P*
Time in PACU (min)	79.93	17.65	75.22	18.21	0.2945
Time in second-stage recovery (min)	70.07	18.52	68.44	19.10	0.7269
Time from PACU to discharge (min)	159.56	25.99	152.89	26.80	0.3086
First PACU VAS pain score	6.59	1.30	6.67	1.34	0.8161
Final PACU VAS pain score	4.18	0.83	4.11	0.86	0.7469
Final VAS pain score prior to discharge	3.97	0.81	4.27	0.83	0.1467
Average VAS pain score	4.83	0.74	4.92	0.77	0.6225
Pain level on follow-up call	2.26	0.96	2.47	0.99	0.3746

Means are predicted values evaluated with covariates at 0.

*P*-value <0.05 is in **Bold**.

### Other factors

Age was not a significant factor in levels of preoperative VAS pain scores, first PACU VAS pain score, final PACU VAS pain score, final VAS pain score prior to discharge, average postoperative VAS pain score nor VAS pain score on follow-up call. Females showed an increase in first PACU VAS pain score (6.05 versus 5.15 *P* = 0.0026), final PACU VAS pain score, (4.43 versus 3.90, *P* = 0.0045), final VAS pain score prior to discharge (3.87 versus 3.03, *P* < 0.001) and average postoperative pain score (4.60 versus 4.03, *P* < 0.001) compared to male patients, but no difference in VAS pain score on follow-up call (2.44 versus 2.49, *P* = 0.36) (Table VII).

**Table VII.** Other characteristics of the study population that may influence postoperative pain including age, sex and smoking status.

*Pain factors*	*Age*				*Sex*				
*Pain variable*	*n*	*Slope*	*R2*	*P*	*n*	*Female mean*	*N*	*Male mean*	*P*
Preoperative VAS pain score	37	0.0387	0.0063	0.374	37	2.892	0	–	–
First PACU VAS pain score	408	−0.0225	0.0227	0.098	262	6.05	145	5.145	0.0026
Final PACU VAS pain score	396	−0.0105	0.0067	0.209	256	4.434	139	3.899	0.0045
Final VAS pain score prior to discharge	407	−0.0084	0.004	0.298	261	3.874	145	3.028	<0.001
Average postoperative VAS pain score	408	−0.0138	0.0027	0.075	262	4.793	145	4.033	<0.001
VAS pain score on follow-up call	326	−0.0138	0.0078	0.152	217	2.684	108	2.486	0.3595

*P*-value <0.05 is in **Bold**.

## DISCUSSION

Gabapentin in the preoperative period does not appear to significantly impact duration of time in postoperative recovery areas nor did it have any significant association with the postoperative VAS pain score. The patients who received ketorolac intraoperatively, although they also received more intraoperative and total MME, did not demonstrate any significant association with the change in time in postoperative recovery areas nor did it demonstrate a difference with postoperative VAS pain scores. Age was not a significant factor; however, being female was significant for having higher VAS pain scores. Several of these findings warrant further discussion.

These results are consistent with those in the study by Ul Huda *et al.* who demonstrated that gabapentin did not decrease postoperative pain following shoulder arthroscopy [12] and with a systematic review by Hannon *et al.* who showed no decrease in postoperative pain after hip arthroplasty [18]. Cunningham *et al.* found that ketorolac was associated with a decrease in opioids following hip arthroscopy for FAI [17]; however, we did not see these same effects. This is likely due to differences in anesthesia providers as we saw an increase in intraoperative MME utilization for the intraoperative ketorolac group and an increase in preoperative MME in the preoperative gabapentin group, but no increase in postoperative opioid consumption in either group. We contribute these differences in the MME to preferences of the anesthesia providers and if they prefer to give additional medications preoperatively or intraoperatively.

We found that female sex was associated with higher postoperative VAS pain scores, thus suggesting that patient factors are an important factor in postoperative pain control. Munsch *et al.* also described potential factors relating to postoperative pain when they described increased age being associated with increased pain [9]. However, our study did not have these same results, but both findings highlight the importance of patient factors when discussing postoperative pain and the many contributing variables.

None of the study participants received peripheral nerve blocks. However, all participants received an intra-articular and periportal injection of 20cc of ropivacaine at the conclusion of each procedure. This could potentially explain the lack of significant differences between the experimental groups. Kim *et al.* demonstrated in a recent systematic review and meta-analysis that local anesthetic was just as effective as peripheral nerve blocks at treating postoperative pain by comparing opioid use and VAS scores [19]. The local anesthetic used in this study could blunt the differences if any or confound the results of the gabapentin and ketorolac analysis.

Limitations of this study include the retrospective review of the data and the lack of an established protocol for administration of the medications. Another limitation to this study is the possibility of type II error due to the small sample size in the non-gabapentin group. In an effort to maintain consistency, only primary hip scopes with femoroplasty and acetabuloplasty were included for treatment of FAI. All revisions and other procedures were excluded to decrease confounding variables. In regard to medication dosing, study design and analysis were limited due to the retrospective nature of the study and different anesthesia providers as well as the paucity of literature on standard of care for hip arthroscopy analgesia. Medication dosing was at the discretion of the anesthesia providers but included 30 mg of ketorolac unless it was weight-based dosing for patients less than 18 years old. Gabapentin dosing was typically given at 300 or 600 mg, but other doses were included due to the small sample size. Dosing could be related to patient size or other factors. This study was specifically looking at immediate postoperative pain. It is also worth considering any long-term trends that may develop in the days to weeks after surgical procedures. The state this study was conducted in has a prescription database for controlled substances. That could be used for future studies regarding pain medication usage following hip arthroscopy.

A subset of participants in this study received both gabapentin and ketorolac, which may be synergistic or confounding. The authors acknowledge that these are potential confounding variables with both treatments. We aimed to minimize these by performing subset analysis of both treatments separately. There is literature such as comparing local anesthesia to fascia iliaca blocks [8], preoperative NSAID administration [15] and cold therapy [9] but no consensus on ideal perioperative pain management for hip arthroscopy.

## CONCLUSION

From the findings in this study, we found that preoperative gabapentin or intraoperative ketorolac did not significantly impact postoperative VAS pain scores or time to discharge from the hospital after hip arthroscopy. These results suggest that although there may be theoretical advantages of these medications in the perioperative period, those results were not present in our study.

## Data Availability

The data underlying this article will be shared on reasonable request to the corresponding author.

## Level of Evidence

III
